# First Report on the Whitefly, *Aleurodicus pseudugesii* on the Coconut Palm, *Cocos nucifera* in Brazil

**DOI:** 10.1673/031.012.2601

**Published:** 2012-02-18

**Authors:** Rose Paula Mendonça de Omena, Elio Cesar Guzzo, Joana Maria Santos Ferreira, Fernando Antônio Cavalcante de Mendonça, Aurino Florencio de Lima, Francisco Racca-Filho, Antônio Euzébio Goulart Santana

**Affiliations:** ^1^ Universidade Federal de Alagoas, Av. Lourival Melo Mota, s/n, Tabuleiro do Martins, 7 57072-970, Maceió, AL, Brasil; ^2^ Embrapa Tabuleiros Costeiros/UEP Alagoas, Cx Postal 2013, 57061-970, Maceió, AL, Brasil; ^3^ Embrapa Tabuleiros Costeiros, Cx Postal 44, 49001-970, Aracaju, SE, Brasil; ^4^ Universidade do Estado da Bahia, R. da Gangorra, 503 Bairro General Dutra, 48600-000, Paulo Afonso, BA, Brasil; ^5^ Universidade Federal Rural do Rio de Janeiro, BR 465 km 7, CEP, 23890-000, Seropédica, RJ, Brasil

**Keywords:** pest, Hemiptera, Sternorrhyncha, Aleyrodidae

## Abstract

The coconut palm, *Cocos nucifera* L. (Arecales: Arecaceae), is currently grown extensively throughout the intertropical zones of the world, including Brazil, where it constitutes an important source of income for growers. Although whiteflies are not normally considered coconut pests, these insects can damage crops directly by sucking the sap, which weakens the plant; indirect damage may be caused by sooty mold formation over the excreted honeydew and by the transmission of pathogens. Whiteflies have infested coconut plants in the northeastern, northern, and southeastern regions of Brazil. Infested materials were collected and the causative insect was identified as *Aleurodicus pseudugesii* Martin (Hemiptera: Aleyrodidae). This is the first report of *A. pseudugesii* in Brazil as a pest of the coconut palm.

## Introduction

The coconut palm, *Cocos nucifera* L. (Arecales: Arecaceae), is one of the 20 most important plant species (Howard 2001a). Currently, it is commercially grown in over 90 countries, covering a total area of approximately 12 million hectares. Mexico and Brazil are the major producers of coconut in the Americas (Killmann 2001; FAO 2011).

One of the limiting factors of coconut palm cultivation is pest attack. Sucking insects, although not usually included in the lists of key pests (Ferreira 2009; Gallo et al. 2002), can become important pests because the direct damage they cause to the palms. They also secrete sugary substances that serve as a substrate for sooty mold (*Capnodium* spp.), which in turn reduces the photosynthetic capacity of the plant, and thereby decreases plant productivity. Sucking insects can transmit phytovirus to important crops. Among the sucking insects, whiteflies are of great agricultural importance because they attack numerous crops worldwide (Gallo et al. 2002) and can reduce crop production by up to 80% (Yuki 2001).

The first species of whitefly reported to attack coconut palms was *Aleurodicus cocois,* which affected coconut plantations on the island of Barbados (Howard 2001b). To date, about 47 species of Aleyrodidae that attack *C. nucifera* have been reported (Howard 2001b; Evans 2007), and of these 17 species were found in Brazil. The purpose of this study is to report for the first time in Brazil the occurrence of *Aleurodicus pseudugesii* Martin (Hemiptera: Aleyrodidae) on coconut palm plantations.

## Materials and Methods

Whiteflies were found infesting coconut palms in several commercial plantations in the Brazilian states of Alagoas, Bahia, Ceará, Paraíba, and Sergipe in the northeastern region, Pará in the northern region, and Rio de Janeiro in the southeastern region. Samples of *C. nucifera* were collected from each of these areas. Infested leaves were collected with pruning shears and packed in paper bags. From this material, herbarium specimens containing eggs, nymphs, pupae, and adult insects were segregated and sent to the Entomology Laboratory, Federal Rural University of Rio de Janeiro (Universidade Federal Rural do Rio de Janeiro (UFRRJ)), for species identification. The identification was based on the pupal exuviae, which were mounted on microscope slides with Hoyer's medium and observed under an optical microscope.

After identification, the material was deposited in the Angelo Moreira da Costa Lima Entomological Collection of UFRRJ in Seropédica, Rio de Janeiro.

## Results and Discussion

The whitefly species found on the *C. nucifera* trees was identified as *A. pseudugesii,* which was described from material collected in Ecuador, with paratypes in Peru on the same host species (Martin 2008). This is the first report on the occurrence of *A. pseudugesii* in Brazil, and whether this species is endemic to Brazil, was introduced into Brazil, or was introduced to Ecuador and Peru from Brazil is uncertain.

According to Howard (2001b), Sternorrhyncha is the most widely represented suborder of Hemiptera on palms. Evans (2007) and Howard (2001b) cited 86 species of Aleyrodidae affecting palm trees, of which 47 species affect *C. nucifera.* In Brazil, 19 Aleyrodidae species have reportedly affected palm trees. Seventeen of these species affect coconut palms and 15 are Aleurodicinae (Table 1). The reason why such a large number of Aleyrodidae species infect *C. nucifera* is not necessarily because this palm is a preferred host species or is more suitable for insects, but because it is cultivated as a monoculture in all tropical regions of the world. In addition, the pests are monitored much more frequently for coconut palms than any other palm (Howard 2001b).

Whitefly adults usually do not disperse far from the leaf on which they developed; most dispersal is to other leaves of the same plant or neighboring plants. However, adults can make long distance dispersal flights of over 7 km where they depend primarily on air currents to cover long distances (Byrne 1999). Alighting at the end of dispersal flights is guided primarily by their attraction to yellow— green wavelengths of light (Blackmer et al. 1995), which is not plant specific; consequently, they readily land on host and non—host plants alike.

**Figure 1.**
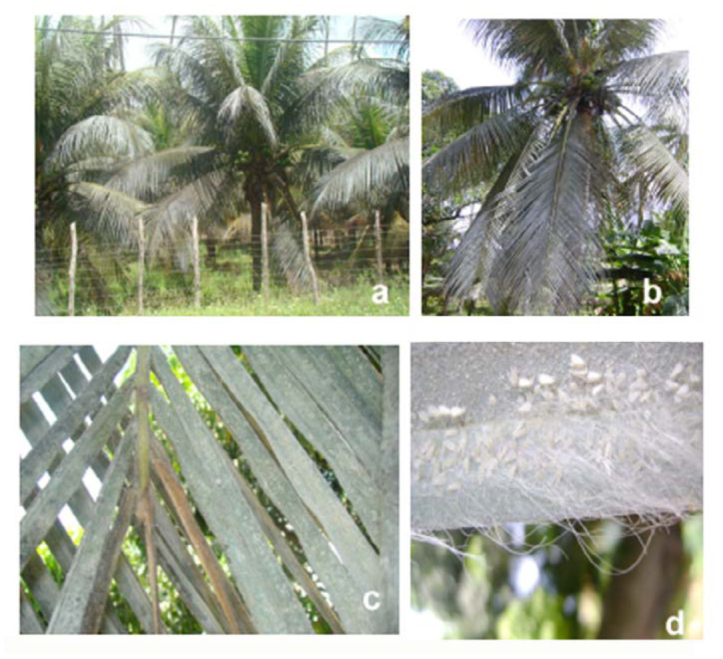
*Aleurodicus pseudugesii* on *Cocos nucifera.* (a, b) — Leaves with a silvery appearance, which is a characteristic of the insect attack; (c, d) colonies of insects covered with a serous secretion and white filaments. High quality figures are available online.

Mere ovipositioning of Aleyrodidae on a plant does not necessarily characterize the plant as a host. However, when an Aleyrodidae species reaches the pupa stage on a plant, it is likely to complete its development on that plant until adulthood. Because the identification of whiteflies is based on the characteristics of its pupal exuviae, which is a sessile form during insect development, designation of a plant species as a host is reliable (Howard 2001b).

The signs of an *A. pseudugesii* attack on coconut palms are very characteristic. The leaves on the plant's crown acquire a silver tone (Figure 1a and 1b). Individual insects focus on the lower leaves of the plant and can colonize entire leaves. They preferentially harbor on the ventral surface of leaflets where they can produce very high—density colonies, covered with serous fluid and white filaments (Figure 1c and 1d). Sooty molds are often associated with such attacks.

Infestations of whiteflies are harmful to crops because mycelia of the fungus (sooty mold) *Capnodium* spp. form a dense and dark layer on the plant surface preventing sunlight from reaching the photosynthetic tissue, and thus reducing photosynthesis (Howard 2001b; Gallo et al. 2002; Anderson 2005; Morales 2005). Furthermore, the continuous suction of the sap drains the plant's energy, removing essential nutrients required for its growth and reproduction, which leads to a reduced crop productivity (Howard 2001b; Gallo et al. 2002; Anderson 2005; Morales 2005). In addition, the whiteflies transmit more than 100 viral diseases to plants, though only a few species have been recognized as vectors, and none of these diseases have been found in palms yet (Jones 2003).

Although whiteflies are commonly found infesting coconut and other palms, they usually occur at low densities. Even the heaviest infestations occur sporadically. Hence, their real importance is often overshadowed by other pests that cause more evident damage. According to Howard (2001b), this is the reason why whiteflies have not been mentioned in the lists of important pests affecting coconut palms in the main producing regions of the world.

To protect itself from adverse environmental conditions such as intense sunlight and heavy rains (Howard 2001a), *A. pseudugesii* colonies are concentrated on the lower surface of leaflets and on coconut leaves that are woven together to form a mat. Despite these protective measures, a reduced insect infestation was observed after the heavy rains last April in the areas studied.

Further studies should be conducted to determine the actual extent of *A. pseudugesii* distribution in Brazil, their potential as pests of coconut palms, control methods, etc.

**Table 1.**
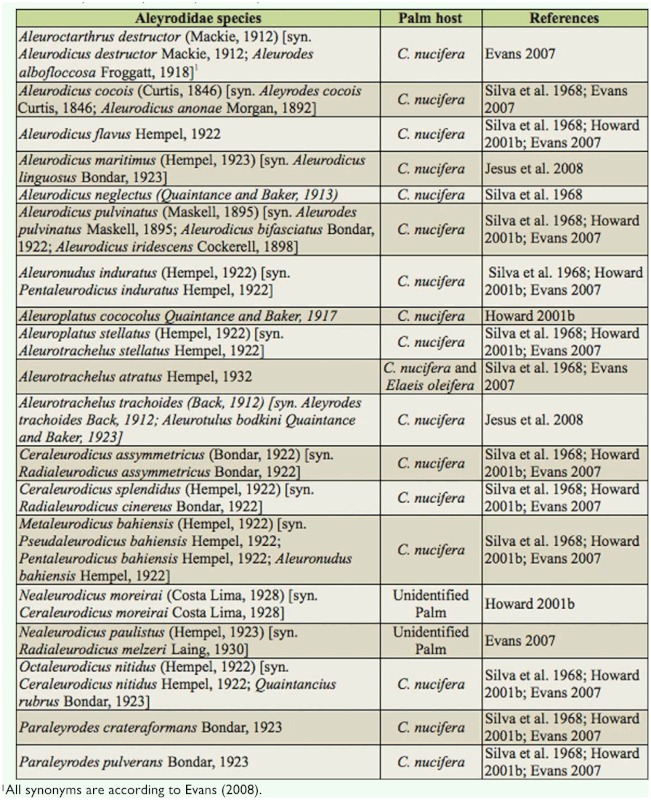
Aleyrodidae species reported on palms in Brazil.
